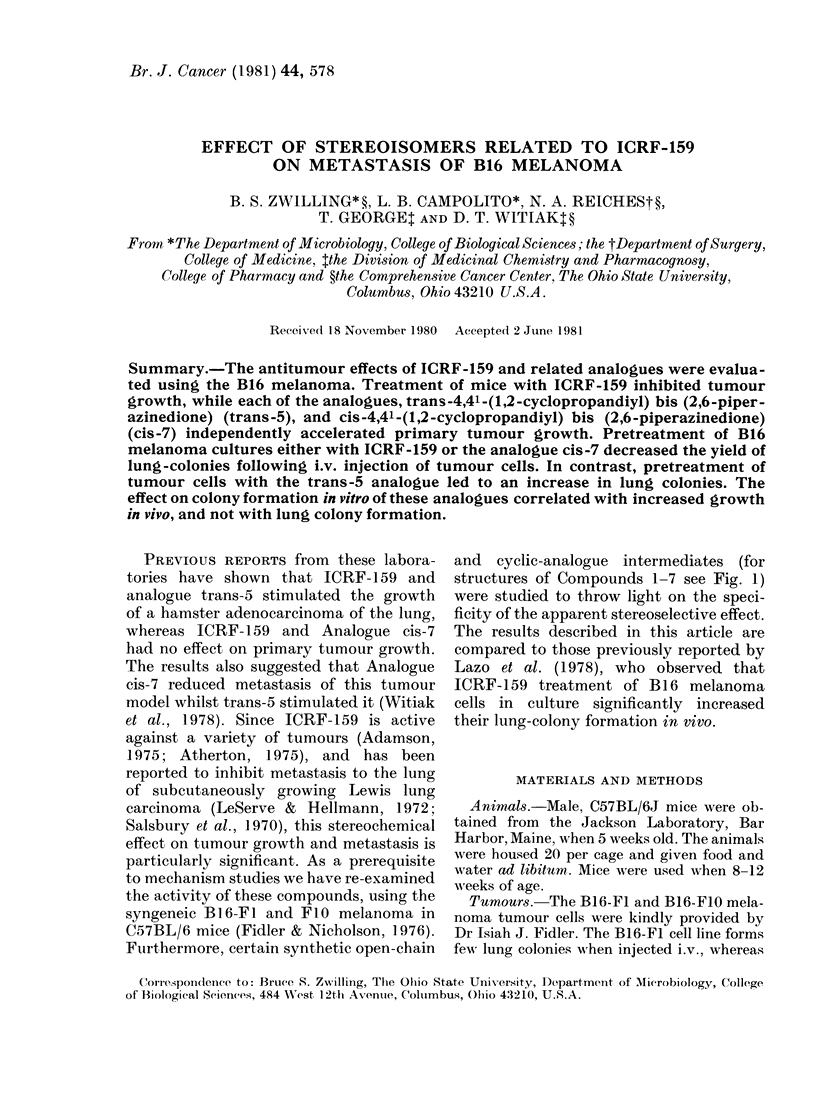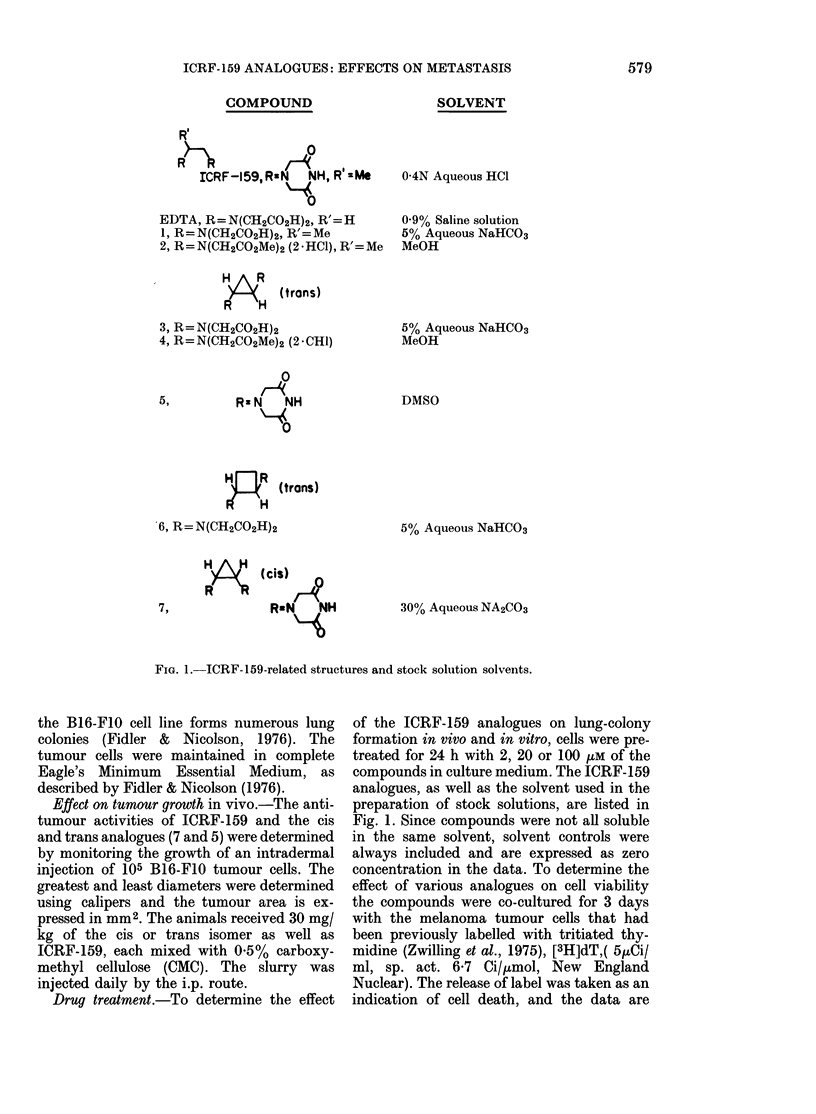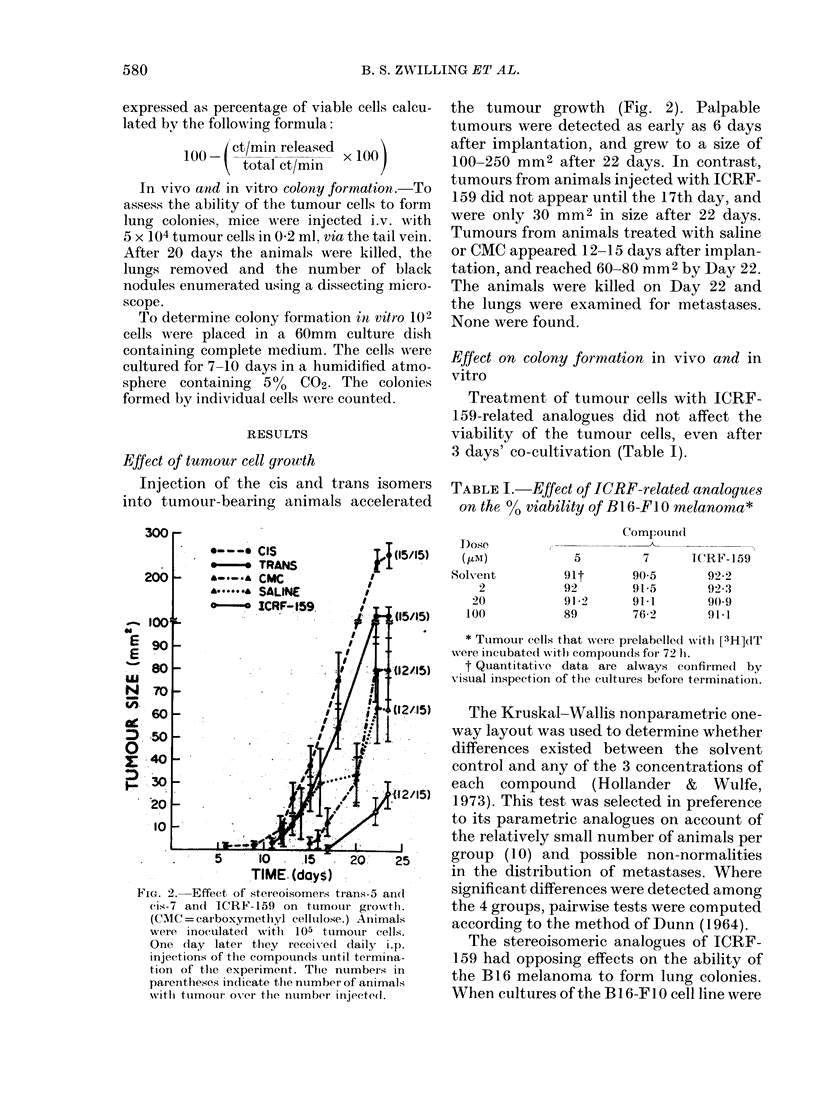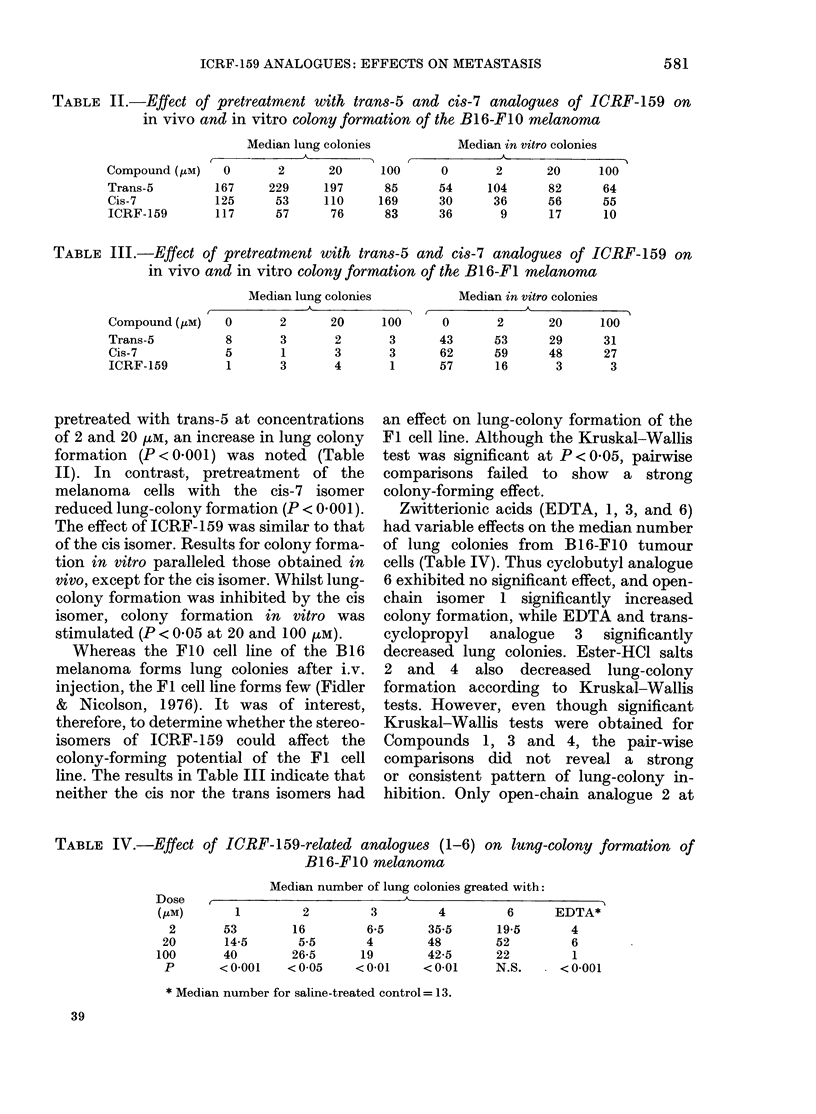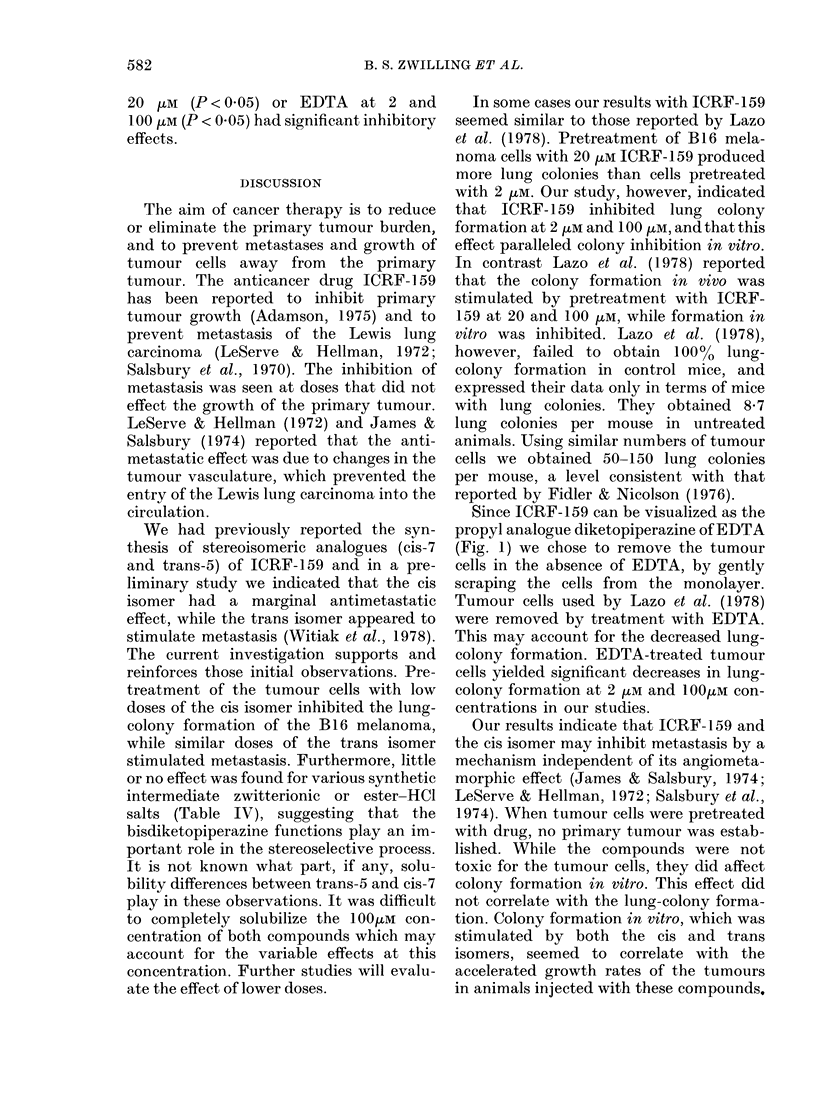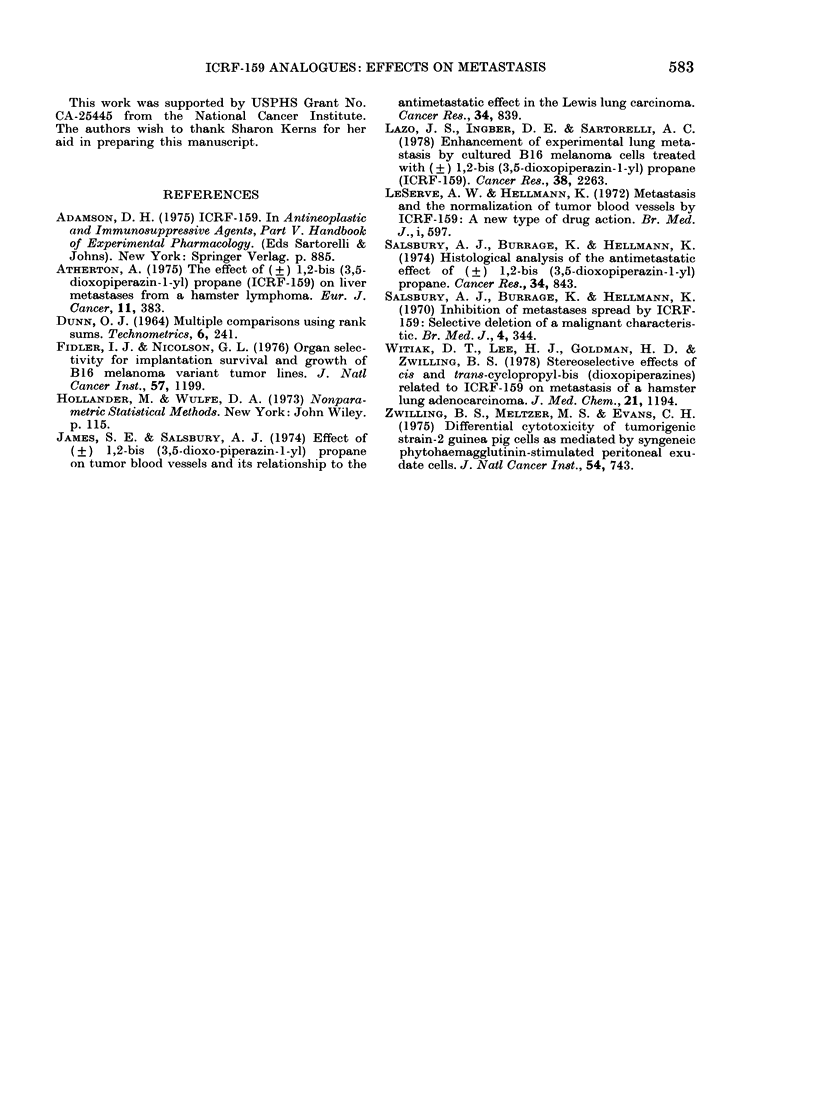# Effect of stereoisomers related to ICRF-159 on metastasis of B16 melanoma.

**DOI:** 10.1038/bjc.1981.229

**Published:** 1981-10

**Authors:** B. S. Zwilling, L. B. Campolito, N. A. Reiches, T. George, D. T. Witiak

## Abstract

The antitumour effects of ICRF-159 and related analogues were evaluated using the B16 melanoma. Treatment of mice with ICRF-159 inhibited tumour growth, while each of the analogues, trans-4,4(1)-(1,2-cyclopropandiyl) bis (2,6-piperazinedione) (trans-5), and cis-4,4(1)-(1,2-cyclopropandiyl) bis (2,6-piperazinedione) (cis-7) independently accelerated primary tumour growth. Pretreatment of B16 melanoma cultures either with ICRF-159 or the analogue cis-7 decreased the yield of lung-colonies following i.v. injection of tumour cells. In contrast, pretreatment of tumour cells with the trans-5 analogue led to an increase in lung colonies. The effect on colony formation in vitro of these analogues correlated with increased growth in vivo, and not with lung colony formation.


*Br. J. Cancer* (1981) **44**, 578

### EFFECT OF STEREOISOMERS RELATED TO ICRF-159 ON METASTASIS OF B16 MELANOMA

B. S. ZWILLING*§, L. B. CAMPOLITO*, N. A. REICHES†§, T. GEORGE‡ AND D. T. WITIAK‡§

*From *The Department of Microbiology, College of Biological Sciences; the †Department of Surgery, College of Medicine, ‡the Division of Medicinal Chemistry and Pharmacognosy, College of Pharmacy and §the Comprehensive Cancer Center, The Ohio State University, Columbus, Ohio* 43210 *U.S.A.*

Received 18 November 1980 Accepted 2 June 1981

**Summary.—The antitumour effects of ICRF-159 and related analogues were evaluated using the B16 melanoma. Treatment of mice with ICRF-159 inhibited tumour growth, while each of the analogues, trans-4,4¹-(1,2-cyclopropandiyl) bis (2,6-piperazinedione) (trans-5), and cis-4,4¹-(1,2-cyclopropandiyl) bis (2,6-piperazinedione) (cis-7) independently accelerated primary tumour growth. Pretreatment of B16 melanoma cultures either with ICRF-159 or the analogue cis-7 decreased the yield of lung-colonies following i.v. injection of tumour cells. In contrast, pretreatment of tumour cells with the trans-5 analogue led to an increase in lung colonies. The effect on colony formation *in vitro* of these analogues correlated with increased growth *in vivo*, and not with lung colony formation.**

PREVIOUS REPORTS from these laboratories have shown that ICRF-159 and analogue trans-5 stimulated the growth of a hamster adenocarcinoma of the lung, whereas ICRF-159 and Analogue cis-7 had no effect on primary tumour growth. The results also suggested that Analogue cis-7 reduced metastasis of this tumour model whilst trans-5 stimulated it (Witiak *et al.*, 1978). Since ICRF-159 is active against a variety of tumours (Adamson, 1975; Atherton, 1975), and has been reported to inhibit metastasis to the lung of subcutaneously growing Lewis lung carcinoma (LeServe & Hellmann, 1972; Salsbury *et al.*, 1970), this stereochemical effect on tumour growth and metastasis is particularly significant. As a prerequisite to mechanism studies we have re-examined the activity of these compounds, using the syngeneic B16-F1 and F10 melanoma in C57BL/6 mice (Fidler & Nicholson, 1976). Furthermore, certain synthetic open-chain and cyclic-analogue intermediates (for structures of Compounds 1–7 see Fig. 1) were studied to throw light on the specificity of the apparent stereoselective effect. The results described in this article are compared to those previously reported by Lazo *et al.* (1978), who observed that ICRF-159 treatment of B16 melanoma cells in culture significantly increased their lung-colony formation *in vivo*.

### MATERIALS AND METHODS

*Animals.*—Male, C57BL/6J mice were obtained from the Jackson Laboratory, Bar Harbor, Maine, when 5 weeks old. The animals were housed 20 per cage and given food and water *ad libitum*. Mice were used when 8–12 weeks of age.

*Tumours.*—The B16-F1 and B16-F10 melanoma tumour cells were kindly provided by Dr Isiah J. Fidler. The B16-F1 cell line forms few lung colonies when injected i.v., whereas

Correspondence to: Bruce S. Zwilling, The Ohio State University, Department of Microbiology, College of Biological Sciences, 484 West 12th Avenue, Columbus, Ohio 43210, U.S.A.

| COMPOUND | SOLVENT |
|---|---|
|   | 0·4N Aqueous HCl |
| EDTA, R = $N(CH_2CO_2H)_2$, R′ = H | 0·9% Saline solution |
| 1, R = $N(CH_2CO_2H)_2$, R′ = Me | 5% Aqueous $NaHCO_3$ |
| 2, R = $N(CH_2CO_2Me)_2$ (2·HCl), R′ = Me | MeOH |
|   | |
| 3, R = $N(CH_2CO_2H)_2$ | 5% Aqueous $NaHCO_3$ |
| 4, R = $N(CH_2CO_2Me)_2$ (2·CHl) | MeOH |
| 5,   | DMSO |
|   | |
| 6, R = $N(CH_2CO_2H)_2$ | 5% Aqueous $NaHCO_3$ |
| 7,   | 30% Aqueous $NA_2CO_3$ |

FIG. 1.—ICRF-159-related structures and stock solution solvents.

the B16-F10 cell line forms numerous lung colonies (Fidler & Nicolson, 1976). The tumour cells were maintained in complete Eagle's Minimum Essential Medium, as described by Fidler & Nicolson (1976).

*Effect on tumour growth* in vivo.—The anti-tumour activities of ICRF-159 and the cis and trans analogues (7 and 5) were determined by monitoring the growth of an intradermal injection of $10^5$ B16-F10 tumour cells. The greatest and least diameters were determined using calipers and the tumour area is expressed in $mm^2$. The animals received 30 mg/kg of the cis or trans isomer as well as ICRF-159, each mixed with 0·5% carboxy-methyl cellulose (CMC). The slurry was injected daily by the i.p. route.

*Drug treatment.*—To determine the effect of the ICRF-159 analogues on lung-colony formation *in vivo* and *in vitro*, cells were pre-treated for 24 h with 2, 20 or 100 $\mu$M of the compounds in culture medium. The ICRF-159 analogues, as well as the solvent used in the preparation of stock solutions, are listed in Fig. 1. Since compounds were not all soluble in the same solvent, solvent controls were always included and are expressed as zero concentration in the data. To determine the effect of various analogues on cell viability the compounds were co-cultured for 3 days with the melanoma tumour cells that had been previously labelled with tritiated thymidine (Zwilling *et al.*, 1975), [$^3$H]dT,( 5$\mu$Ci/ml, sp. act. 6·7 Ci/$\mu$mol, New England Nuclear). The release of label was taken as an indication of cell death, and the data are expressed as percentage of viable cells calculated by the following formula:

$$100 - \left(\frac{\text{ct/min released}}{\text{total ct/min}} \times 100\right)$$

In vivo *and* in vitro *colony formation.*—To assess the ability of the tumour cells to form lung colonies, mice were injected i.v. with $5 \times 10^4$ tumour cells in 0·2 ml, *via* the tail vein. After 20 days the animals were killed, the lungs removed and the number of black nodules enumerated using a dissecting microscope.

To determine colony formation *in vitro* $10^2$ cells were placed in a 60mm culture dish containing complete medium. The cells were cultured for 7–10 days in a humidified atmosphere containing 5% $CO_2$. The colonies formed by individual cells were counted.

### RESULTS

### *Effect of tumour cell growth*

Injection of the cis and trans isomers into tumour-bearing animals accelerated the tumour growth (Fig. 2). Palpable tumours were detected as early as 6 days after implantation, and grew to a size of 100–250 mm² after 22 days. In contrast, tumours from animals injected with ICRF-159 did not appear until the 17th day, and were only 30 mm² in size after 22 days. Tumours from animals treated with saline or CMC appeared 12–15 days after implantation, and reached 60–80 mm² by Day 22. The animals were killed on Day 22 and the lungs were examined for metastases. None were found.

FIG. 2.—Effect of stereoisomers trans-5 and cis-7 and ICRF-159 on tumour growth. (CMC = carboxymethyl cellulose.) Animals were inoculated with $10^5$ tumour cells. One day later they received daily i.p. injections of the compounds until termination of the experiment. The numbers in parentheses indicate the number of animals with tumour over the number injected.

### *Effect on colony formation* in vivo *and* in vitro

Treatment of tumour cells with ICRF-159-related analogues did not affect the viability of the tumour cells, even after 3 days' co-cultivation (Table I).

TABLE I.—*Effect of ICRF-related analogues on the % viability of B16-F10 melanoma**

| Dose (μM) | Compound 5 | 7 | ICRF-159 |
|---|---|---|---|
| Solvent | 91† | 90·5 | 92·2 |
| 2 | 92 | 91·5 | 92·3 |
| 20 | 91·2 | 91·1 | 90·9 |
| 100 | 89 | 76·2 | 91·1 |

* Tumour cells that were prelabelled with [³H]dT were incubated with compounds for 72 h.

† Quantitative data are always confirmed by visual inspection of the cultures before termination.

The Kruskal–Wallis nonparametric one-way layout was used to determine whether differences existed between the solvent control and any of the 3 concentrations of each compound (Hollander & Wulfe, 1973). This test was selected in preference to its parametric analogues on account of the relatively small number of animals per group (10) and possible non-normalities in the distribution of metastases. Where significant differences were detected among the 4 groups, pairwise tests were computed according to the method of Dunn (1964).

The stereoisomeric analogues of ICRF-159 had opposing effects on the ability of the B16 melanoma to form lung colonies. When cultures of the B16-F10 cell line were

TABLE II.—*Effect of pretreatment with trans-5 and cis-7 analogues of ICRF-159 on in vivo and in vitro colony formation of the B16-F10 melanoma*

| | Median lung colonies | | | | Median *in vitro* colonies | | | |
|---|---|---|---|---|---|---|---|---|
| Compound (μM) | 0 | 2 | 20 | 100 | 0 | 2 | 20 | 100 |
| Trans-5 | 167 | 229 | 197 | 85 | 54 | 104 | 82 | 64 |
| Cis-7 | 125 | 53 | 110 | 169 | 30 | 36 | 56 | 55 |
| ICRF-159 | 117 | 57 | 76 | 83 | 36 | 9 | 17 | 10 |

TABLE III.—*Effect of pretreatment with trans-5 and cis-7 analogues of ICRF-159 on in vivo and in vitro colony formation of the B16-F1 melanoma*

| | Median lung colonies | | | | Median *in vitro* colonies | | | |
|---|---|---|---|---|---|---|---|---|
| Compound (μM) | 0 | 2 | 20 | 100 | 0 | 2 | 20 | 100 |
| Trans-5 | 8 | 3 | 2 | 3 | 43 | 53 | 29 | 31 |
| Cis-7 | 5 | 1 | 3 | 3 | 62 | 59 | 48 | 27 |
| ICRF-159 | 1 | 3 | 4 | 1 | 57 | 16 | 3 | 3 |

pretreated with trans-5 at concentrations of 2 and 20 μM, an increase in lung colony formation ($P < 0{\cdot}001$) was noted (Table II). In contrast, pretreatment of the melanoma cells with the cis-7 isomer reduced lung-colony formation ($P < 0{\cdot}001$). The effect of ICRF-159 was similar to that of the cis isomer. Results for colony formation *in vitro* paralleled those obtained *in vivo*, except for the cis isomer. Whilst lung-colony formation was inhibited by the cis isomer, colony formation *in vitro* was stimulated ($P < 0{\cdot}05$ at 20 and 100 μM).

Whereas the F10 cell line of the B16 melanoma forms lung colonies after i.v. injection, the F1 cell line forms few (Fidler & Nicolson, 1976). It was of interest, therefore, to determine whether the stereoisomers of ICRF-159 could affect the colony-forming potential of the F1 cell line. The results in Table III indicate that neither the cis nor the trans isomers had an effect on lung-colony formation of the F1 cell line. Although the Kruskal–Wallis test was significant at $P < 0{\cdot}05$, pairwise comparisons failed to show a strong colony-forming effect.

Zwitterionic acids (EDTA, 1, 3, and 6) had variable effects on the median number of lung colonies from B16-F10 tumour cells (Table IV). Thus cyclobutyl analogue 6 exhibited no significant effect, and open-chain isomer 1 significantly increased colony formation, while EDTA and trans-cyclopropyl analogue 3 significantly decreased lung colonies. Ester-HCl salts 2 and 4 also decreased lung-colony formation according to Kruskal–Wallis tests. However, even though significant Kruskal–Wallis tests were obtained for Compounds 1, 3 and 4, the pair-wise comparisons did not reveal a strong or consistent pattern of lung-colony inhibition. Only open-chain analogue 2 at

TABLE IV.—*Effect of ICRF-159-related analogues (1–6) on lung-colony formation of B16-F10 melanoma*

| Dose | Median number of lung colonies greated with: | | | | | |
|---|---|---|---|---|---|---|
| (μM) | 1 | 2 | 3 | 4 | 6 | EDTA* |
| 2 | 53 | 16 | 6·5 | 35·5 | 19·5 | 4 |
| 20 | 14·5 | 5·5 | 4 | 48 | 52 | 6 |
| 100 | 40 | 26·5 | 19 | 42·5 | 22 | 1 |
| *P* | < 0·001 | < 0·05 | < 0·01 | < 0·01 | N.S. | < 0·001 |

* Median number for saline-treated control = 13.

20 $\mu$M ($P < 0{\cdot}05$) or EDTA at 2 and 100 $\mu$M ($P < 0{\cdot}05$) had significant inhibitory effects.

### DISCUSSION

The aim of cancer therapy is to reduce or eliminate the primary tumour burden, and to prevent metastases and growth of tumour cells away from the primary tumour. The anticancer drug ICRF-159 has been reported to inhibit primary tumour growth (Adamson, 1975) and to prevent metastasis of the Lewis lung carcinoma (LeServe & Hellman, 1972; Salsbury *et al.*, 1970). The inhibition of metastasis was seen at doses that did not effect the growth of the primary tumour. LeServe & Hellman (1972) and James & Salsbury (1974) reported that the anti-metastatic effect was due to changes in the tumour vasculature, which prevented the entry of the Lewis lung carcinoma into the circulation.

We had previously reported the synthesis of stereoisomeric analogues (cis-7 and trans-5) of ICRF-159 and in a preliminary study we indicated that the cis isomer had a marginal antimetastatic effect, while the trans isomer appeared to stimulate metastasis (Witiak *et al.*, 1978). The current investigation supports and reinforces those initial observations. Pretreatment of the tumour cells with low doses of the cis isomer inhibited the lung-colony formation of the B16 melanoma, while similar doses of the trans isomer stimulated metastasis. Furthermore, little or no effect was found for various synthetic intermediate zwitterionic or ester–HCl salts (Table IV), suggesting that the bisdiketopiperazine functions play an important role in the stereoselective process. It is not known what part, if any, solubility differences between trans-5 and cis-7 play in these observations. It was difficult to completely solubilize the 100$\mu$M concentration of both compounds which may account for the variable effects at this concentration. Further studies will evaluate the effect of lower doses.

In some cases our results with ICRF-159 seemed similar to those reported by Lazo *et al.* (1978). Pretreatment of B16 melanoma cells with 20 $\mu$M ICRF-159 produced more lung colonies than cells pretreated with 2 $\mu$M. Our study, however, indicated that ICRF-159 inhibited lung colony formation at 2 $\mu$M and 100 $\mu$M, and that this effect paralleled colony inhibition *in vitro*. In contrast Lazo *et al.* (1978) reported that the colony formation *in vivo* was stimulated by pretreatment with ICRF-159 at 20 and 100 $\mu$M, while formation *in vitro* was inhibited. Lazo *et al.* (1978), however, failed to obtain 100% lung-colony formation in control mice, and expressed their data only in terms of mice with lung colonies. They obtained 8·7 lung colonies per mouse in untreated animals. Using similar numbers of tumour cells we obtained 50–150 lung colonies per mouse, a level consistent with that reported by Fidler & Nicolson (1976).

Since ICRF-159 can be visualized as the propyl analogue diketopiperazine of EDTA (Fig. 1) we chose to remove the tumour cells in the absence of EDTA, by gently scraping the cells from the monolayer. Tumour cells used by Lazo *et al.* (1978) were removed by treatment with EDTA. This may account for the decreased lung-colony formation. EDTA-treated tumour cells yielded significant decreases in lung-colony formation at 2 $\mu$M and 100$\mu$M concentrations in our studies.

Our results indicate that ICRF-159 and the cis isomer may inhibit metastasis by a mechanism independent of its angiometamorphic effect (James & Salsbury, 1974; LeServe & Hellman, 1972; Salsbury *et al.*, 1974). When tumour cells were pretreated with drug, no primary tumour was established. While the compounds were not toxic for the tumour cells, they did affect colony formation *in vitro*. This effect did not correlate with the lung-colony formation. Colony formation *in vitro*, which was stimulated by both the cis and trans isomers, seemed to correlate with the accelerated growth rates of the tumours in animals injected with these compounds.

This work was supported by USPHS Grant No. CA-25445 from the National Cancer Institute. The authors wish to thank Sharon Kerns for her aid in preparing this manuscript.

### REFERENCES

ADAMSON, D. H. (1975) ICRF-159. In *Antineoplastic and Immunosuppressive Agents, Part V. Handbook of Experimental Pharmacology*. (Eds Sartorelli & Johns). New York: Springer Verlag. p. 885.

ATHERTON, A. (1975) The effect of (±) 1,2-bis (3,5-dioxopiperazin-1-yl) propane (ICRF-159) on liver metastases from a hamster lymphoma. *Eur. J. Cancer*, **11**, 383.

DUNN, O. J. (1964) Multiple comparisons using rank sums. *Technometrics*, **6**, 241.

FIDLER, I. J. & NICOLSON, G. L. (1976) Organ selectivity for implantation survival and growth of B16 melanoma variant tumor lines. *J. Natl Cancer Inst.*, **57**, 1199.

HOLLANDER, M. & WULFE, D. A. (1973) *Nonparametric Statistical Methods*. New York: John Wiley. p. 115.

JAMES, S. E. & SALSBURY, A. J. (1974) Effect of (±) 1,2-bis (3,5-dioxo-piperazin-1-yl) propane on tumor blood vessels and its relationship to the antimetastatic effect in the Lewis lung carcinoma. *Cancer Res.*, **34**, 839.

LAZO, J. S., INGBER, D. E. & SARTORELLI, A. C. (1978) Enhancement of experimental lung metastasis by cultured B16 melanoma cells treated with (±) 1,2-bis (3,5-dioxopiperazin-1-yl) propane (ICRF-159). *Cancer Res.*, **38**, 2263.

LESERVE, A. W. & HELLMANN, K. (1972) Metastasis and the normalization of tumor blood vessels by ICRF-159: A new type of drug action. *Br. Med. J.*, i, 597.

SALSBURY, A. J., BURRAGE, K. & HELLMANN, K. (1974) Histological analysis of the antimetastatic effect of (±) 1,2-bis (3,5-dioxopiperazin-1-yl) propane. *Cancer Res.*, **34**, 843.

SALSBURY, A. J., BURRAGE, K. & HELLMANN, K. (1970) Inhibition of metastases spread by ICRF-159: Selective deletion of a malignant characteristic. *Br. Med. J.*, **4**, 344.

WITIAK, D. T., LEE, H. J., GOLDMAN, H. D. & ZWILLING, B. S. (1978) Stereoselective effects of *cis* and *trans*-cyclopropyl-bis (dioxopiperazines) related to ICRF-159 on metastasis of a hamster lung adenocarcinoma. *J. Med. Chem.*, **21**, 1194.

ZWILLING, B. S., MELTZER, M. S. & EVANS, C. H. (1975) Differential cytotoxicity of tumorigenic strain-2 guinea pig cells as mediated by syngeneic phytohaemagglutinin-stimulated peritoneal exudate cells. *J. Natl Cancer Inst.*, **54**, 743.